# Development of Multi-Scale Carbon Nanofiber and Nanotube-Based Cementitious Composites for Reliable Sensing of Tensile Stresses

**DOI:** 10.3390/nano12010074

**Published:** 2021-12-28

**Authors:** Shama Parveen, Bruno Vilela, Olinda Lagido, Sohel Rana, Raul Fangueiro

**Affiliations:** 1 Department of Fashion and Textiles, School of Arts and Humanities, University of Huddersfield, Huddersfield HD1 3DH, UK; 2 Department of Civil Engineering, University of Minho, Campus de Azurém, 4800-058 Guimarães, Portugal; bmnvilela@gmail.com (B.V.); olinda.lagido@live.com.pt (O.L.); 3 Department of Mechanical Engineering, University of Minho, Campus de Azurém, 4800-058 Guimarães, Portugal

**Keywords:** multi-scale composites, carbon fibers, cement, carbon nanotubes, stress sensing

## Abstract

In this work, multi-scale cementitious composites containing short carbon fibers (CFs) and carbon nanofibers (CNFs)/multi-walled carbon nanotubes (MWCNTs) were studied for their tensile stress sensing properties. CF-based composites were prepared by mixing 0.25, 0.5 and 0.75 wt.% CFs (of cement) with water using magnetic stirring and Pluronic F-127 surfactant and adding the mixture to the cement paste. In multi-scale composites, CNFs/MWCNTs (0.1 and 0.15 wt.% of cement) were dispersed in water using Pluronic F-127 and ultrasonication and CFs were then added before mixing with the cement paste. All composites showed a reversible change in the electrical resistivity with tensile loading; the electrical resistivity increased and decreased with the increase and decrease in the tensile load/stress, respectively. Although CF-based composites showed the highest stress sensitivity among all specimens at 0.25% CF content, the fractional change in resistivity (FCR) did not show a linear correlation with the tensile load/stress. On the contrary, multi-scale composites containing CNFs (0.15% CNFs with 0.75% CFs) and MWCNTs (0.1% MWCNTs with 0.5% CFs) showed good stress sensitivity, along with a linear correlation between FCR and tensile load/stress. Stress sensitivities of 6.36 and 11.82%/MPa were obtained for the best CNF and MWCNT-based multi-scale composite sensors, respectively.

## 1. Introduction

Cementitious composites are extensively used in civil infrastructures and are susceptible to deterioration of their properties over time. Therefore, health monitoring of cement-based buildings and infrastructures at periodic intervals is an important requirement to ensure the safety of the occupants, as well as to extend the lifespan of the infrastructures. The monitoring of real-time conditions and performance of structures, which is known as structural health monitoring (SHM), is performed mainly in the critical zones of the structures using various sensors [1,2]. The collected data are used to evaluate the health conditions of structures in order to take timely maintenance actions. SHM is frequently performed using various sensors such as optical fiber sensors, electrical resistance strain gauges, piezoelectric (PZT) ceramics, etc., each one of which has their own limitations [1,2] and, consequently, a great deal of research is currently underway to find an affordable, reliable and easy-to-use technique for SHM of civil infrastructures.

From past few years, investigations on the piezoresistive cementitious composites (i.e., composites which show change in their electrical resistivity with mechanical stress/strain) for SHM applications have accelerated considerably [1,2,3]. Piezoresistive cementitious sensors have better compatibility with civil structures and are durable [1,2,3]. These sensors were initially developed using short carbon fibers (SCFs) [4,5]. However, researchers are currently utilizing various electrically conductive nanofillers to introduce piezoresistivity into cementitious composites [6,7,8]. Nanomaterials are preferred over carbon fibers (CFs), as they are required at much lower concentrations and provide a positive influence on other properties of cementitious composites (e.g., mechanical properties, microstructure, thermal properties, etc.) due to their high surface area and aspect (i.e., length/diameter) ratio [9,10,11]. Extensive studies have been carried out to date on developing piezoresistive cementitious composites using different nanomaterials such as multi-wall carbon nanotubes (MWCNTs), graphene, nano graphite platelets, spiky spherical nickel powders containing nano tip, carbon nano fibers (CNF), nano carbon black (NCB), etc. [6,12,13].

Formation of a percolating electrical network is required to achieve piezoresistive properties in cementitious composites. Percolation threshold is defined as the critical concentration of conductive fillers to enable non-conductive cementitious matrices to show conductivity [7]. The percolation threshold of CFs (5 mm long and 10 µm diameter) within a cementitious matrix was found to be between 0.5 (~0.3 wt.%) and 1 vol.% (~0.58 wt.%) [4,5]. The percolation threshold of MWCNT in cementitious composites was also found in the similar range (between 0.3 and 0.6 wt.% of cement) [14]. However, according to Yoo et al., the optimum concentration of MCNTs to introduce piezoresistivity into cementitious matrices was found to be 1 wt.% (with respect to cement) [7,8]. For graphene-based cementitious composites, the percolation threshold was found to be between 1 and 5 wt.% of graphene and the resulting composites showed good piezoresistive properties [12]. However, MWCNTs were considered as superior and more effective nanofillers for the fabrication of piezoresistive cementitious composites when compared to graphite nanofibers and graphene when used in similar concentrations [8]. More recently, the use of hybrid conductive fillers, i.e., the combination of two different fillers proved more effective in achieving superior conductivity and sensing properties in cementitious materials (these composites are known as multi-scale composites, as they are developed using hybrid reinforcements with micro- and nano-scale diameters [9]). For example, the use of CFs (15 mm in length and 5–7 µm in diameter) in combination with MWCNTs improved the stability of electrical resistivity of cementitious composites [14]. Reliable sensing of compressive loads and strains of cementitious composites was also achieved with hybrid conductive fillers composed of CFs and MWCNTs [15]. Cementitious composites containing these hybrid fillers demonstrated superior repeatability of sensing results when compared to CF-based cementitious composites [14]. Zhang et al. recently reported that a hybrid filler system containing CNT/NCB (40:60) showed a percolation threshold of 0.39 to 1.49 vol.% (of mortar) and the resulting cementitious composites demonstrated a stable and sensitive piezoresistive property [16]. The observed percolation threshold and piezoresistivity of CNTs (and other nanomaterials), CFs and hybrid fillers in cementitious composites were different in different studies and this was attributed to the use of different types of nanomaterials and CFs (possessing different diameters and aspect ratios), their dispersion states, as well as different cement/water ratios and compositions used for the development of cementitious composites. Besides piezoresistivity, the hybrid filles were also found to be effective in improving the physical and mechanical properties of cementitious composites. For example, the use of 0.25 wt.% SCFs (of cement) with 0.75 wt.% of MWCNTs (of cement) improved the flexural strength by ~243%, flexural modulus by 200% and toughness by 672% of plain cement-based composites [17]. Also, cementitious composites with 2.25 wt.% SCF and 0.5 wt.% MWCNTs improved the tensile strength of plain cement composites by ~53%, tensile modulus by 60% and failure strain by 44% [18].

Due to the growing interest and prospect of piezoresistive cementitious composites in the civil engineering sector, a cost-effective and an easy fabrication method to develop these composites is highly desirable. Although cementitious composites containing hybrid fillers showed superior results, only a few studies have been carried out to date. Also, to the best of the authors’ knowledge, the existing studies investigated the sensing properties of hybrid cementitious composites under compression loading and no study has been conducted to date under tensile loading mode. The present study, therefore, investigated and compared the piezoresistive properties of CF-reinforced, CF-MWCNT and CF-CNF hybrid filler-reinforced cementitious composites under tensile loading. CNFs and MWCNTs were selected as the conductive filles for developing these hybrid cementitious composites due to their high electrical conductivity (possessing electrical resistivity as low as 1 × 10^−4^ and 2 × 10^−3^ − 1 × 10^−4^ Ωcm, respectively), relatively low cost when compared to other conductive nanomaterials such as single-walled CNTs, graphene, etc., high aspect ratio (250–2000 and 100–10,000, respectively), as well as their high mechanical properties (tensile strength of 2.92 and 10–60 GPa, respectively and tensile modulus of 240 and 1000 GPa, respectively] [19]. The comparison of the piezoresistive behavior of CF-MWCNT and CF-CNF hybrid filler-reinforced cementitious composites has also not been addressed in the existing literature. Moreover, a non-ionic surfactant, Pluronic F-127, was used for the first time to ensure proper dispersion of the fillers in the developed sensing cementitious composites. The piezoresistive properties of the composites were studied under cyclic tensile loading at different loading conditions. The fractional change in resistivity (FCR), its correlation with the applied load and stress sensitivity were determined and discussed in detail.

## 2. Materials and Methods

### 2.1. Raw Materials

**Figure 1:**
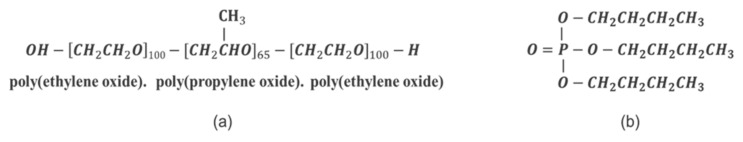
Chemical structure of (**a**) Pluronic F-127 and (**b**) Tri butyl Phosphate.

Cementitious composite specimens were fabricated using the Portland cement CEM I 42.5 R (purchased by Lisbon, Secil, Portugal). The properties of this cement are summarized in Table 1. Short CFs (Tenax^®^, diameter: 7.0 µm, Length: 5 mm) were supplied by Teijin Carbon Europe GmbH (Wuppertal, Germany) and MWCNTs and CNFs were supplied by Nanostructured & Amorphous Materials, Inc. (Houston, TX, USA). Their physical and mechanical properties are summarized in Table 2. CNFs had an electrical conductivity of more than 100 S/cm. CNFs may contain significant amount of amorphous carbon, as well as residual catalysts and other inorganic impurities such as Fe, Co, S, etc. and a trace amount of Mg, Cl, Ca, Cr, etc. [20] and these residual catalyst particles and other impurities can significantly influence the electrical conductivity of CNFs. Pluronic F-127 (the chemical structure is provided in Figure 1a), a non-ionic surfactant, was used to disperse MWCNTs/CNFs and CFs in water and was purchased from Sigma Aldrich (Algés, Portugal). A defoamer, tri-butyl phosphate (the chemical structure is provided in Figure 1b), was supplied by Acros Organics (Thermo Fischer Scientific, Porto Salvo, Portugal).

### 2.2. Characterization of Morphology of Carbon Nanofibers and Nanotubes

Scanning Electron Microscopy (FEG-SEM, NOVA 200 Nano SEM, FEI, Hillsboro, Oregon, USA) at an acceleration voltage of 10 kV was used to study the morphology of MWCNTs and CNFs. To avoid the charging of samples during SEM, they were coated with a 30 nm film of Au-Pd in a high-resolution sputter coater (208HR Cressington, Watford, UK).

### 2.3. Preparation of Aqueous Suspensions of Carbon Fibers, Carbon Nanofibers and Nanotubes

**Figure 2:**
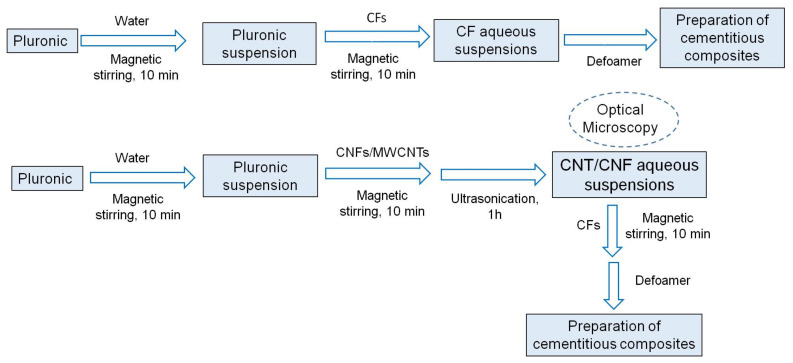
Schematic diagram showing the preparation of aqueous suspensions for fabrication of cementitious composites.

**Figure 3:**
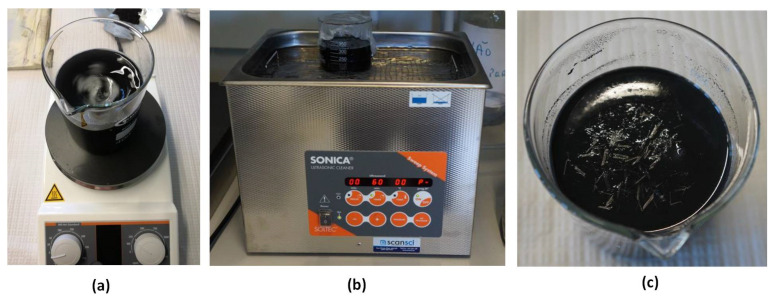
Preparation of aqueous suspensions: (**a**) magnetic stirring, (**b**) ultrasonication and (**c**) the aqueous suspension containing CFs and MWCNTs.

The schematic diagram, showing the preparation of various aqueous suspensions, is shown in Figure 2. The aqueous suspensions of CF, using 5 wt.% of Pluronic F-127 (on the weight of water), were prepared by first mixing Pluronic F-127 in water with the help of magnetic stirring for 10 min. CFs were then added in the surfactant solution and mixed with the help of magnetic stirring for another 10 min. In case of aqueous suspensions containing CFs, along with MWCNTs or CNFs, MWCNT or CNF powder was first added to the surfactant solution and then magnetic stirring was carried out for 10 min to ensure that there were no big lumps of MWCNTs/CNFs in the aqueous suspensions. The MWCNT/CNF surfactant suspensions were then kept in a bath ultrasonicator (Sonica Ultrasonicator 3200 S3, Milan, Italy) operated at 40 kHz frequency and 180 W power for 1 h. After removing the MWCNT/CNF suspensions from the ultrasonicator, CFs were added and mixed using magnetic stirring for 10 min. The defoamer (in the weight ratio of 1:0.5 with respect to Pluronic F-127) was then added to the suspensions, which were used later for the fabrication of cementitious composites. For the characterization of CNT/CNF dispersion in aqueous suspensions, the defoamer and CFs were not added to avoid film formation by the defoamer and agglomeration caused by CFs during the characterization of aqueous suspensions. Figure 2 shows the magnetic stirring and ultrasonication processes of aqueous suspensions and Figure 3c shows a suspension containing MWCNTs and CFs.

### 2.4. Characterization of Aqueous Suspensions

Optical microscopy (Olympus BH2 Microscope, Hamburg, Germany) was used to identify the CNT/CNF agglomerates in the aqueous suspensions. This characterization was performed to also study the homogeneity of the prepared suspensions. To carry out the optical microscopic analysis, a drop of MWCNT/CNFs (without CF) suspension was placed on a glass slide and covered with a cover slip. The images were captured in 5 different places of the drop. The analysis was repeated for 3 times and the images were captured in two different magnifications to clearly understand the quality of the prepared suspensions.

### 2.5. Preparation of Cementitious Composites

**Figure 4:**
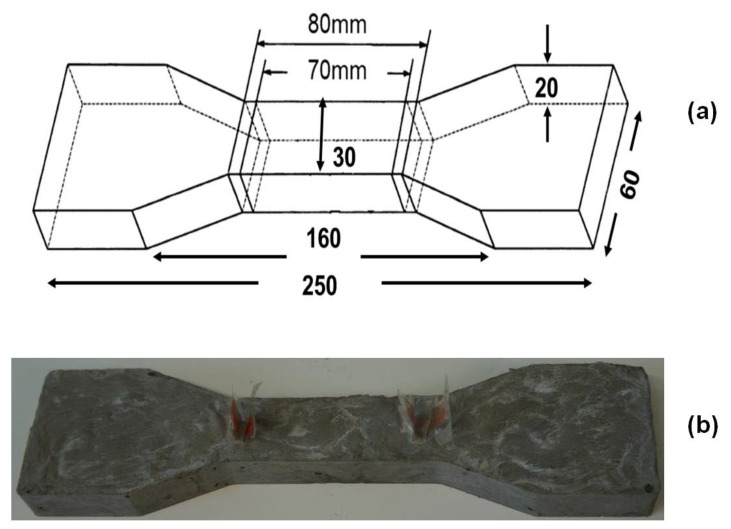
(**a**) Dimension of the dog bone samples. (**b**) Samples used for piezoresistive characterization.

Cementitious composites were fabricated using aqueous suspensions of CF or CF with MWCNTs/CNFs (315 mL) and cement (900 g) following EN 196-1:2006 standard. A set of unreinforced samples, i.e., plain mortars, were also prepared using water (315 mL) to compare with the reinforced cementitious composites. The weighed amount of cement was mixed with the aqueous suspensions using a Hobart mixer; the mixer was set for 1.5 min at a slow speed (140 ± 5 rpm) and then 1.5 min at a high speed (285 ± 10 rpm). The mixtures were then poured into the molds (three samples were prepared for each mixture). The specimens were prepared in a dog-bone shaped mold (having a cross section of 30 mm × 20 mm, the distance between the inner grid for voltage measurement was 70 mm and the outer grid for passing current was 80 mm), as shown in Figure 4, in order to perform the piezoresistive measurement. The grids were made of copper foils having 30 mm × 15 mm dimension.

The molds were placed on a jolting machine for 30 s to remove the entrapped air. The molded samples were then covered by cellophane and placed in a chamber with a moist atmosphere for 24 h. The samples were demolded after 24 h and kept submerged in water for 28 days at 25 °C. The samples were taken out 4 h before the test, wiped with a cotton cloth and kept at the room temperature prior to the piezoresistive characterization. The compositions of different samples prepared are listed in Table 3.

### 2.6. Characterization of Piezoresistive Properties

**Figure 5:**
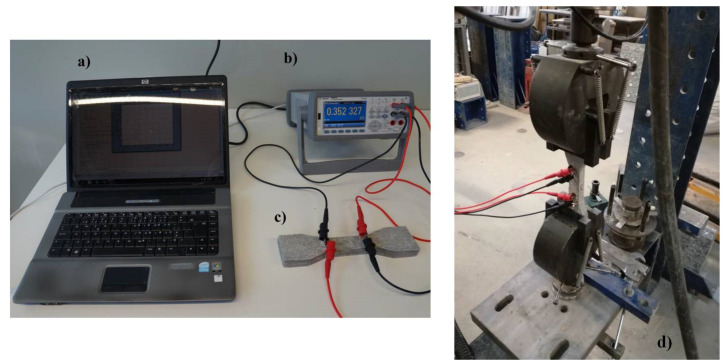
Measurement of (**a**) electrical resistance and (**b**,**c**) piezoresistive property of cementitious composites (**d**) piezoresistive property of cementitious composites in tensile mode.

The test setups for the characterization of the electrical resistance and stress sensing properties of cementitious composites are shown in Figure 5. Stress sensing properties were characterized by measuring the electrical resistivity of samples using a digital multimeter (Agilent 34460a, Santa Clara, CA, USA) in the elastic regime of tensile loading with three different loading rates: 20, 30 and 40 N.s^−1^. For each loading rate, the load was increased from 0 N up to 10 s and then decreased to 0 N at the same unloading rate. 5 cycles of loading and unloading (20 s per cycle) were studied at each loading rate to verify the repeatable performance of the developed cementitious composites. The DC electrical resistance was measured simultaneously during the mechanical testing using a four-probe method. Fractional change in the resistivity (*FCR*) and stress sensitivity of the composites in each cycle were calculated using the following equations:

(1) $$FCR=\frac{final\;resistivity\;\left(\rho \right)-initial\;resistivity\;\left(\rho_{0}\right)}{initial\;resistivity\left(\rho_{0}\right)}$$

(2) $$Stress\;Sensitivity=\frac{100\;x\;FCR}{Applied\;tensile\;stress}$$

### 2.7. Microstructural Characterization of Developed Cementitious Composites

The fractured surfaces of developed specimens were analyzed by using SEM (FEG-SEM, NOVA 200 Nano SEM, FEI) using the secondary electron mode and an acceleration voltage of 10 kV after coating with a thin film (30 nm) of Au-Pd in a high-resolution sputter coater (208HR Cressington, Watford, UK).

## 3. Results

### 3.1. Morphology of Carbon Fibers, Nanofibers and Nanotubes

**Figure 6:**
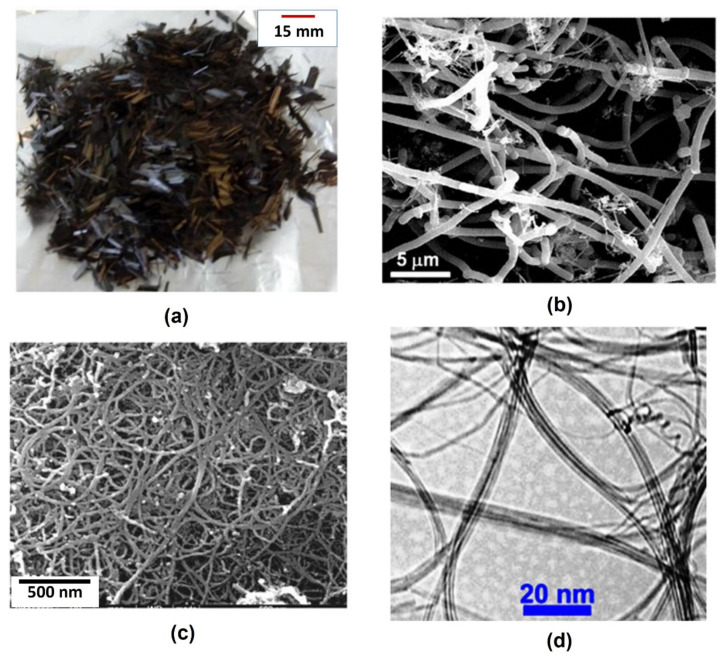
Reinforcement of cementitious composites: (**a**) short CFs, (**b**) SEM micrograph of CNFs and (**c**) SEM micrograph MWCNTs and (**d**) TEM image of MWCNTs, taken from manufacturer website.

The image of short CFs is shown in Figure 6a. The SEM micrographs of CNF and MWCNTs are shown in Figure 6b,c, respectively. Significant entanglements or agglomeration can be observed in the case of CNFs. However, MWCNTs showed the highest degree of agglomeration and clustering in the powder, as can be seen from Figure 6c. Transmission electron microscope (TEM) images of MWCNT aqueous suspensions also showed clustering and entanglements of nanotubes, as shown in Figure 6d. Therefore, to break these MWCNT/CNF agglomerates and disperse them homogeneously within the cementitious matrix, a combination of magnetic stirring (10 min) and ultrasonication (1 h) was used.

### 3.2. Aqueous Suspensions of MWCNT and CNT

**Figure 7:**
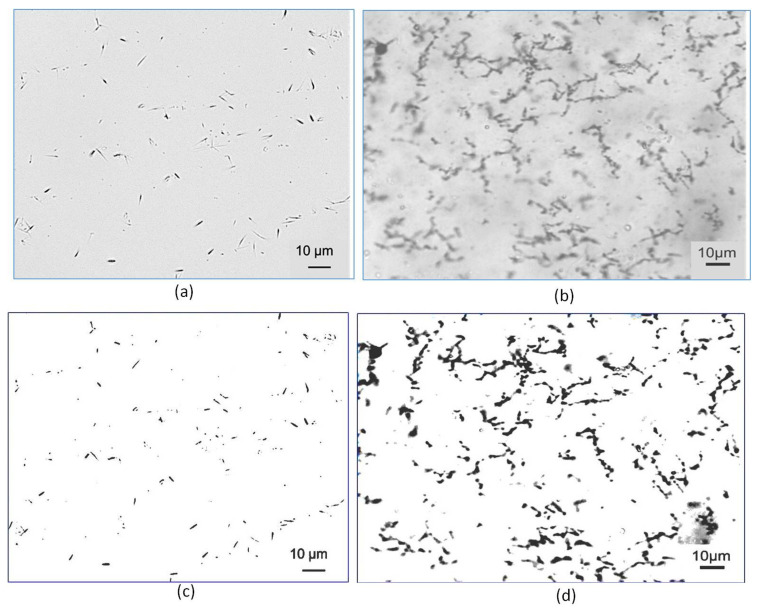
Optical Micrographs of the aqueous suspensions of (**a**) 0.2% MWCNT% with 5 wt.% Pluronic F-127, (**b**) 0.2% CNF with 5 wt.% Pluronic F-127, (**c**,**d**) are the high contrast micrographs of (**a**,**b**), respectively, showing the conductive networks formed by CNFs and MWCNTs.

The optical micrographs of aqueous suspensions of MWCNT and CNF are shown in Figure 7. It is clear from Figure 7a that MWCNTs could be homogeneously dispersed in water using a Pluronic F-127-assisted ultrasonication process. MWCNTs were dispersed without any noticeable agglomeration. Homogeneous dispersion of CNTs and CNFs is prerequisite for developing high performance cementitious composites [9] and the use of Pluronic F-127 was proven to be effective in achieving homogenous CNT dispersion in previous studies also [10,11]. It can also be clearly observed from Figure 7c that dispersed MWCNTs formed electrically conductive pathways within the aqueous medium. The aqueous suspension of CNF also showed homogeneous dispersion free from noticeable CNF agglomerates. To the best of authors’ knowledge, Pluronic F-127 has been utilized to disperse CNFs for the first time in the present study and a homogeneous dispersion was obtained due to the steric stabilization induced by Pluronic F-127 molecules, as previously reported for CNTs [10,11]. However, in this case, a few CNFs were seen to form bundles with each other and formed a greater number of noticeable electrically conductive pathways when compared to MWCNTs within the aqueous medium, as can be seen from Figure 7d.

### 3.3. Electrical Resistance of Cementitious Composites

**Figure 8:**
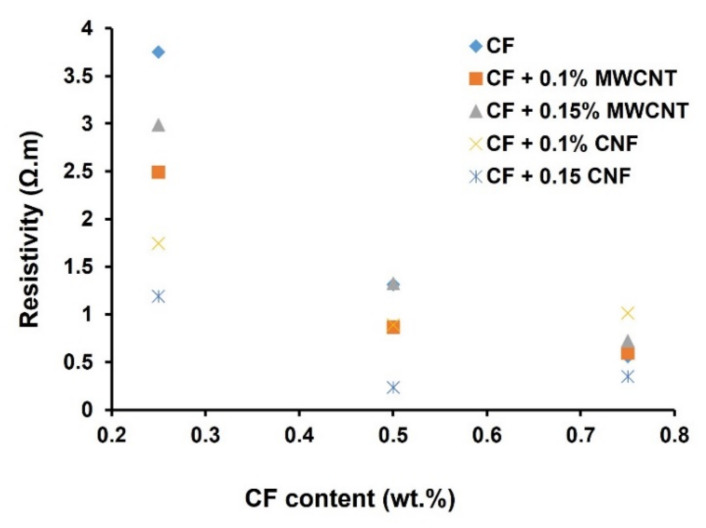
Influence of CF, CNF and MWCNT on the electrical resistance of cementitious composites.

The electrical resistivity of mortar containing only CF and mortar containing CF and different concentrations of CNF and MWCNT is presented in Figure 8. It can be noticed that mortars containing 0.25 wt.% CF had an electrical resistivity of 3.7 Ω.m. The electrical resistivity decreased with the increase in the CF wt.% and the sample containing 0.75 wt.% CF showed an electrical resistivity of 0.6 Ω.m. The electrical resistivity obtained in this case was lower when compared to previously reported mortar samples containing CF, carbon black and other nanomaterials [21,22,23,24].

The use of Pluronic F-127 in the present study was believed to improve the dispersion of CFs within the mortar paste, leading to the reduced clustering of CFs and resulting in a lower porosity of the reinforced cementitious composites. This significantly improved the electrical conductivity of cementitious composites. It is clear from Figure 8 that the addition of MWCNT and CNF significantly reduced the electrical resistivity of CF-reinforced cementitious composites. This was attributed to the fact that the highly conducting MWCNTs and CNFs can bridge the conducting paths formed by CFs and, hence, improved the conductivity of composites [15]. It can also be observed that composites with CNFs at 1.5 wt.% showed a lower resistivity when compared to composites prepared with MWCNTs. This could be due to the better formation of conductive paths with CNFs due to their larger dimensions, as also observed in Figure 7d. Composites with MWCNTs, on the other hand, showed a higher resistivity at the higher concentration, i.e., 1.5 wt.%, probably due to formation of CNT agglomerates, which resulted in the increased porosity and higher resistivity of composites. This observation agrees with the previous studies on CNT-reinforced cementitious composites [25].

### 3.4. Response of Cementitious Composites to Cyclic Tensile Loading

#### 3.4.1. Effect of Cyclic Tensile Loading on Electrical Resistivity

**Figure 9:**
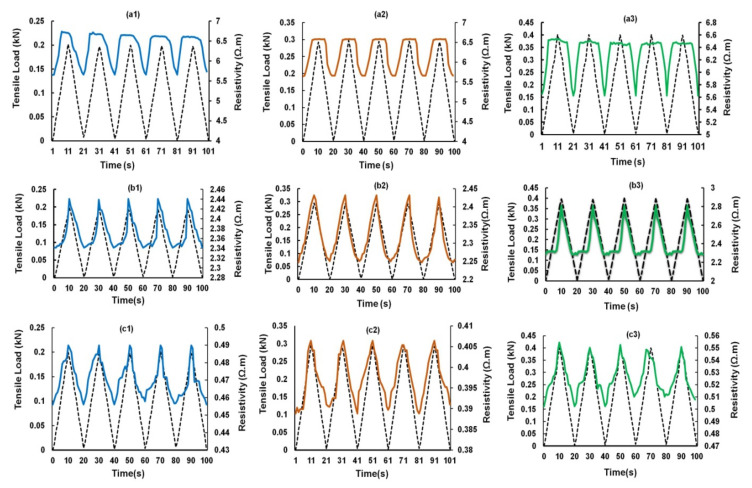
Change of electrical resistance with cyclic tensile load of CF reinforced cementitious composites at different peak loads containing 0.25% CF (**a1**–**a3**), 0.50% CF (**b1**–**b3**) and 0.75% CF (**c1**–**c3**).

The electrical response of CF-reinforced cementitious composites containing 0.25, 0.5 and 0.75 wt.% CF to cyclic tensile loading is shown in Figure 9. These responses were achieved at five loading-unloading cycles at three different loading rates: 20, 30 and 40 N/s. Each loading and unloading cycle took 10 s and therefore, the loading cycles reached 200, 300 and 400 N, respectively, for these three loading rates and came back to 0 N after each unloading cycle. It can be clearly noticed that the electrical resistivity showed a reversible change with the tensile loading, i.e., the electrical resistivity increased with the increase in loading and decreased when the loading decreased in the unloading cycles. The increase in electrical resistivity of short CF-reinforced cementitious composites with increased tensile loading has been previously reported [4,5]. The extension of composites due to tensile loading caused a reduction in the electrical contact points between short CFs, leading to a reduction in the conductive pathways and an increase in the electrical resistivity of composites [4,5]. The change in resistivity in all three studied load levels (i.e., 200, 300 and 400 N) was reversible, indicating that these load levels were within the elastic regime of the composites and did not introduce a permanent damage within the composite structure.

**Figure 10:**
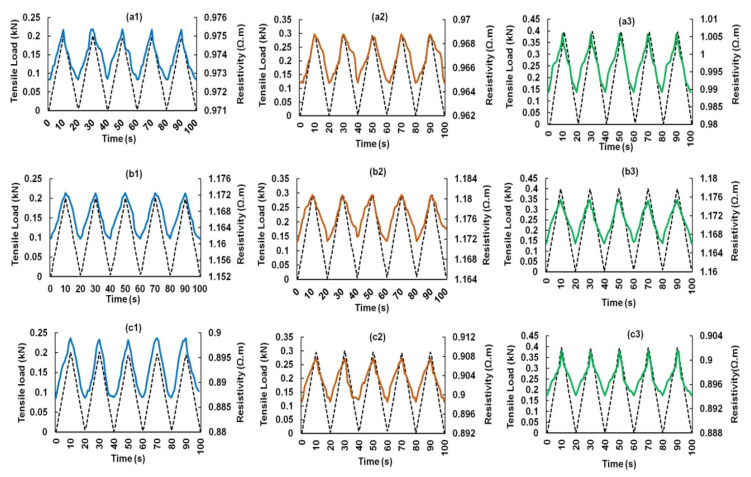
Change of electrical resistance with cyclic tensile load of 0.1% CNF-reinforced cementitious composites at different peak loads containing 0.25% CF (**a1**–**a3**), 0.50% CF (**b1**–**b3**) and 0.75% CF (**c1**–**c3**).

**Figure 11:**
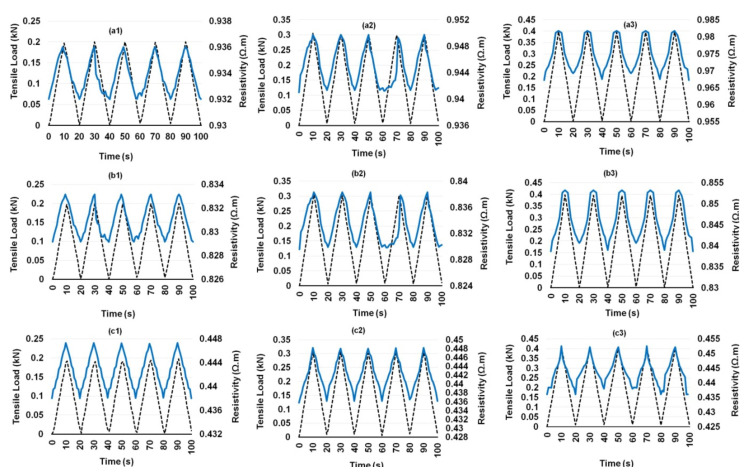
Change of electrical resistance with cyclic tensile load of 0.15% CNF-reinforced cementitious composites at different peak loads containing 0.25% CF (**a1**–**a3**), 0.50% CF (**b1**–**b3**) and 0.75% CF (**c1**–**c3**).

**Figure 12:**
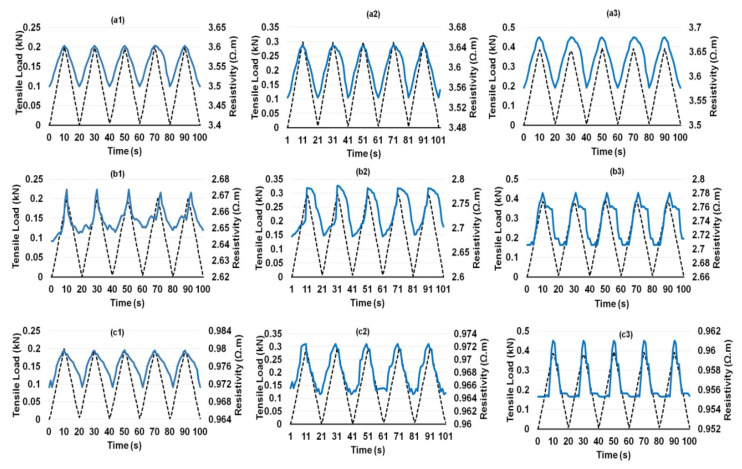
Change of electrical resistance with cyclic tensile load of 0.1% MWCNT-reinforced cementitious composites at different peak loads containing 0.25% CF (**a1**–**a3**), 0.50%SCF (**b1**–**b3**) and 0.75% SCF (**c1**–**c3**).

**Figure 13:**
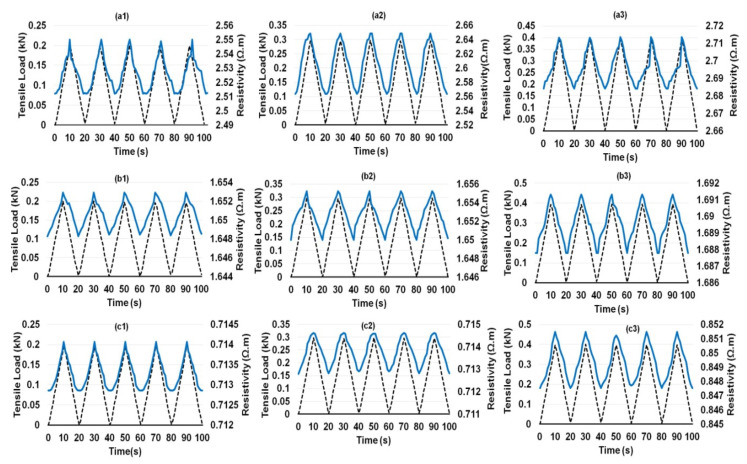
Change of electrical resistance with cyclic tensile load of 0.15% MWCNT-reinforced cementitious composites at different peak loads containing 0.25% CF (**a1**–**a3**), 0.50%SCF (**b1**–**b3**) and 0.75% SCF (**c1**–**c3**).

Composites containing different CF contents showed a similar behavior, except a flattening of electrical resistivity at the maximum load was noticed for the composites containing 0.25 wt.% CF (Figure 9a1–a3). This delayed electrical response from the composites in the region when the loading cycle reversed was probably attributed to a relatively lesser number of electrical contacts between CFs in the case of 0.25 wt.% CF. The electrical response of hybrid cementitious composites containing CF and different concentrations of CNF and MWCNT are shown in Figure 10, Figure 11, Figure 12 and Figure 13. It can be noticed that these composites also showed similar trends of changing their electrical resistivity with tensile loading, i.e., the electrical resistivity increased and decreased reversibly with the increase and decrease in the tensile loading, respectively. However, the extent of electrical resistivity change in different cycles was dependent on the loading rates/maximum load as well as on the composition of the composites.

#### 3.4.2. Fractional Change in Electrical Resistivity and Stress Sensitivity

The average FCR values of five loading cycles for different composites and at different loading conditions are listed in Table 4 and Table 5. It is clear from Table 4 that the average FCR values of CF-reinforced cementitious composites were dependent on the loading rate (and maximum load) and on the amount of CF in these composites.

The influence of the loading rate and maximum load on FCR, however, did not show any clear trend. Previous studies on hybrid nano-carbon containing piezoresistivity cementitious composites showed a decrease in FCR with the increasing rate of compressing loading [13]. This was attributed to the reduced compressive strains with the increasing loading rates, resulting in the reduced resistivity change in each cycle. However, the effect of loading rates on the resistivity change of short CF-based cement composites has not been reported to date. In the present study, both the loading rate and maximum load were changed simultaneously, and this made it difficult to understand their individual effects. An increase in FCR with the increase in the maximum tensile strain per cycle in the elastic regime was previously observed in short CF-based piezoresistive composites [4,5]. In the present case, the increase in loading rates (from 20 to 40 N/s) could decrease the tensile strain of composites, as observed previously in cement-based composites [26]. However, the increase in the maximum load (from 200 to 400 N) could also increase the tensile strain at the same time and, therefore, no clear trend was observed due to these two opposing effects. Further studies by changing the loading rates while maintaining the same maximum load could help to properly understand this phenomenon.

It can also be observed from Table 4 that the FCR values were higher in the case of composites containing 0.25% CFs when compared to composites with 0.5 and 0.75% CFs. CFs formed an electrical percolation network at 0.25% as evidenced by the low resistivity values (see Figure 8). Similar resistivity values were reported previously for CF-based and other cementitious composites above the percolation threshold of the conductive fillers [21,22,23,24]. Therefore, the composites containing 0.25% CFs provided high values of FCR. Previously reported CF-based composites also showed similar FCR values under tensile loading [4,5]. The decrease in FCR at higher CF contents could be attributed to the fact the higher amount of CFs resulted in more touching of CFs and a high number of conductive pathways. This resulted in lower change in the conducting network under tensile deformation and consequently, a lower change in the electrical resistance. This phenomenon was previously observed in the case of CF-reinforced piezoresistive polymeric composites [27,28].

**Figure 14:**
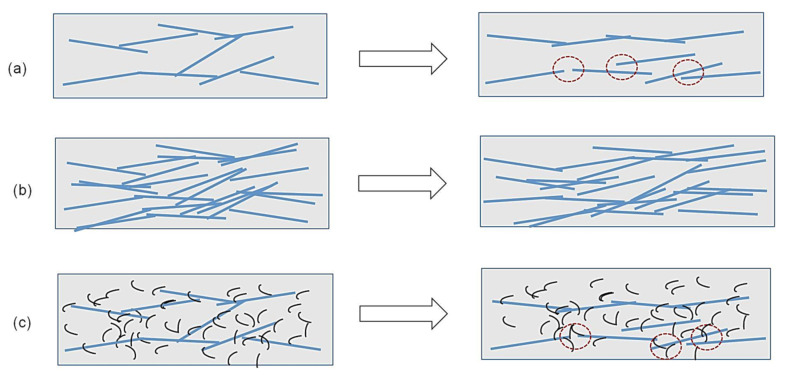
Change in the electorally conductive network under tensile deformation of (**a**) 0.25% CF, (**b**) 0.75% CF and (**c**) 0.25% CF with CNFs/MWCNTs.

It is clear from Table 5 that the cementitious composites with hybrid fillers, i.e., CFs along with CNFs/CNTs showed lower FCR values when compared to only CF-based composites. The reason for this is the same as discussed above for 0.5 and 0.75% CF-based cementitious composites. The presence of CNF and MWCNTs significantly increased the number of conductive pathways and more touching between the conductive fillers, making the conductive network more stable and less sensitive to the mechanical deformations. Figure 14 explains this phenomenon schematically. It can be observed that when the CF content is low (Figure 14a), the conductive network becomes extended under tensile deformation, increasing the distance between CFs (indicated by dotted circles) and leading to a significant increase in the electrical resistivity. However, at higher CF contents, e.g., 0.75% (Figure 14b), due to higher number of electrical contacts the extension of the conductive network does not significantly change the electrical contact points and, therefore, the change in electrical resistivity is limited. Similarly, the presence of MWCNTs/CNFs in the case of 0.25% CF (Figure 14c) can maintain the electrical contacts between CFs when the conductive network extends under tensile deformation (as can be seen in the dotted circles in Figure 14c). Therefore, the presence of MWCNTs/CNFs results in a significantly lower change in the electrical resistivity under tensile loading. Observations made by Kim et al. support this hypothesis as it was noticed that the hybrid fillers composed of CFs and CNTs made the electrically resistivity less sensitive to the change in water to cement ratio, temperature and evaporation of electrolytic pore solution, due to the extended conductive pathways [14]. However, the authors did not evaluate the effect of hybrid fillers on the piezoresistive properties of the composites.

**Figure 15:**
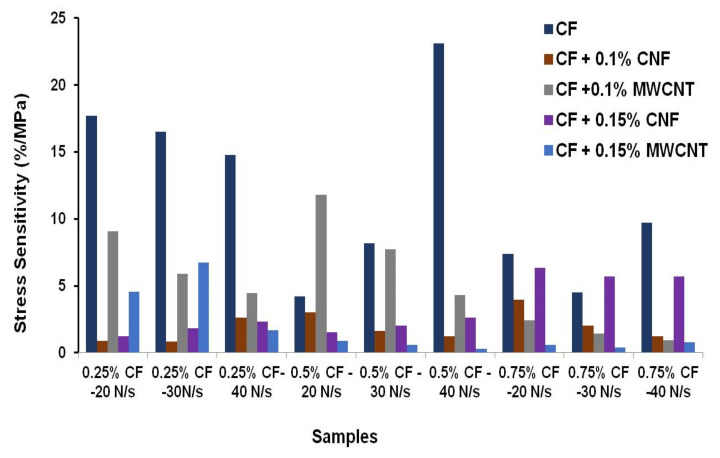
Stress sensitivity of various cement based sensors.

FCR values of cementitious composites containing different concentrations of hybrid fillers are compared in Table 5. It can be noticed that the hybrid composites showed different FCR values depending on the CF, CNF and MWCNT concentrations, as well as the loading conditions, i.e., the rate and maximum load. Similar to CF-based composites, the influence of loading conditions on FCR values did not show any clear trend. The FCR values have been further converted into stress sensitivity according to Equation (2) and are presented in Figure 15. Stress sensitivity is a better parameter to compare the load/stress sensing behavior of cementitious composites containing different concentrations of conductive fillers. It can be observed that the CF-based composites showed a considerably higher stress sensitivity when compared to the hybrid composites due to the higher FCR values of the former, as explained earlier.

In the cases of hybrid composites with 0.25 and 0.5% CF, the use of MWCNTs led to superior stress sensitivity when compared to CNFs. On the contrary, the stress sensitivity was higher with CNFs when compared to MWCNTs when a higher amount of CF, i.e., 0.75 wt.% CF was used in the composites. The superior sensitivity of MWCNT-based composites at lower CF contents could be attributed to the superior ability of MWCNTs to form a piezoresistive network due to their higher electrical conductivity and smaller dimensions. At higher CF contents, a saturation in the electrically conducting pathways resulted in a lower sensitivity of these composites. Also, the stress sensitivity was superior with 0.1% MWCNTs when compared to 0.15% and this could be due to the increased agglomeration of MWCNTs at 0.15% when dispersed using the same process. On the contrary, composites with 0.15% CNF showed superior stress sensitivity when compared to those with 0.1% CNF. This could be attributed to the fact that CNFs could be more homogeneously dispersed at higher concentrations when compared to MWCNTs using the same dispersion process, due to their lower surface area and agglomeration tendency [29].

The highest stress sensitivities achieved with hybrid composites containing 0.15% CNFs and 0.1% CNTs were 6.36 and 11.82%/MPa at 0.75 and 0.5% CF contents, respectively. The observed stress sensitivity values were much higher when compared to those previously reported for hybrid nano-carbon-reinforced cementitious composites under compression loading [13].

### 3.5. FCR-Load Correlations of Developed Sensing Composites

**Figure 16:**
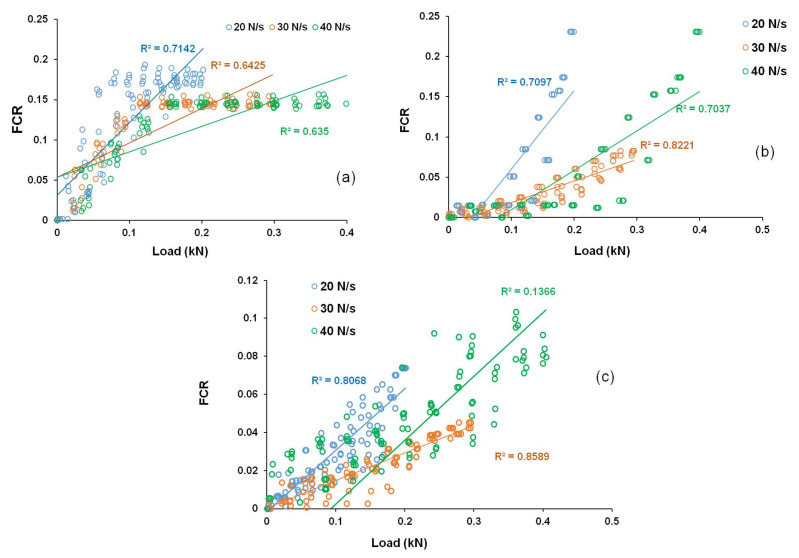
FCR-load correlation of cementitious composites containing different carbon fiber contents: (**a**) 0.25 wt.%, (**b**) 0.5 wt.% and (**c**) 0.75 wt.%.

The relationships between FCR and load for cementitious composites containing different CF contents at different loading conditions are shown in Figure 16. It can be observed that the change in FCR with the tensile load was dependent on the loading conditions. From the values of linear regression coefficients (R^2^), it can be commented that the cementitious composites containing only CFs did not show a good linear correlation between FCR and load (and stress, as stress is proportional to the applied load), making the calibration of these sensors difficult and leading to measurements with high error values. The large scatter of the FCR values indicates an uneven and random change in the electrical resistivity of the samples in different loading and unloading cycles. In CF-based cementitious composites, the loading and unloading cycles caused random and large changes in the electrical contact points between the CFs (schematically shown in Figure 14), making electrical resistivity change and FCR not linearly dependent on the applied load or stress. Further, presence of CF clusters could also lead to unpredictable and random change in the electrical network and, consequently, in the electrical resistivity of the composites. As a result, the scatter in the FCR data was high. As a reliable and accurate measurement with a low scattering of FCR values is required for practical applications, these CF-based sensors are not, therefore, suitable for sensing of tensile stresses in civil engineering structures. A high scatter of FCR values in the case of CF-based cementitious composites was previously observed in the case of compressive loading [15].

**Figure 17:**
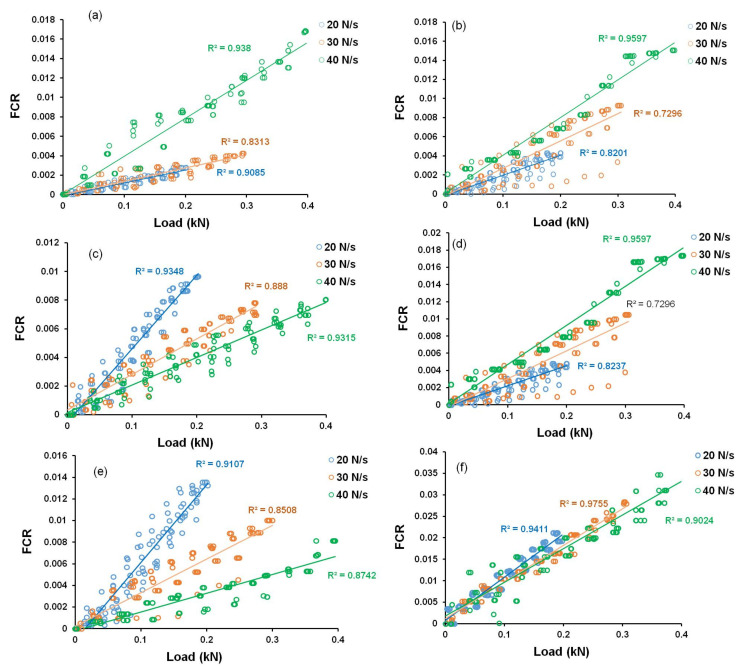
FCR-load correlation of CNF-based hybrid cementitious composites: (**a**) 0.25% CF + 0.1% CNF, (**b**) 0.25% CF + 0.15% CNF, (**c**) 0.5% CF + 0.1% CNF, (**d**) 0.5% CF + 0.15% CNF, (**e**) 0.75% CF + 0.1% CNF and (**f)** 0.75% CF + 0.15% CNF.

The correlation between FCR and tensile load for CNF-based hybrid cementitious composites are shown in Figure 17. It can be observed that hybrid composites containing CNFs presented superior linear correlation between FCR and tensile load (and therefore, with tensile stress) and a lower scatter in FCR values when compared to the composites containing only CFs. It can also be noticed that among different samples, the sample containing 0.15% CNF with 0.75% CFs showed a good linear correlation with a low scatter of data. This composite also presented the best stress sensitivity (6.36%/MPa) and therefore, can be considered as the optimized sample for developing CNF-based hybrid stress sensors for construction applications.

**Figure 18:**
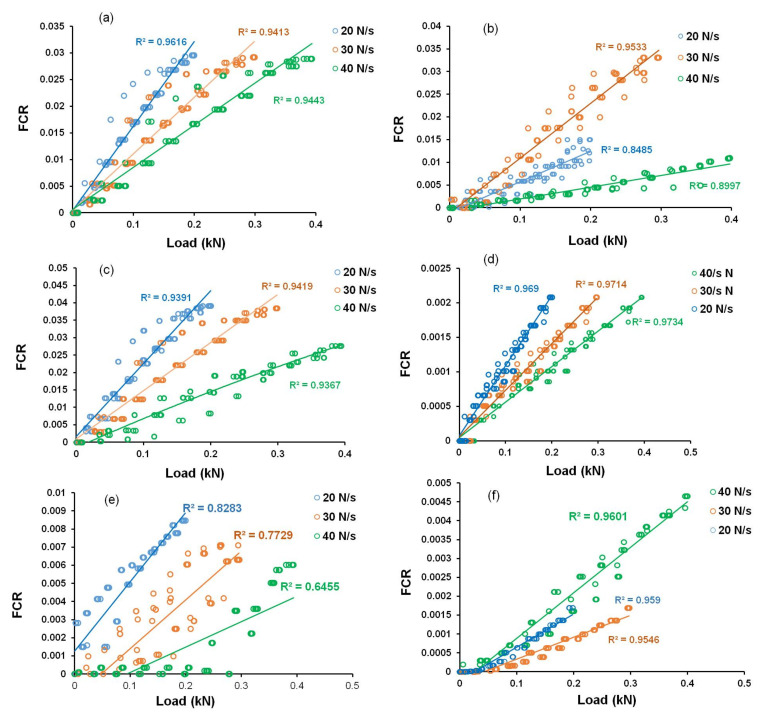
FCR-load correlation of MWCNT-based hybrid cementitious composites: (**a**) 0.25% CF + 0.1% MWCNT, (**b**) 0.25% CF + 0.15% MWCNT, (**c**) 0.5% CF + 0.1% MWCNT, (**d**) 0.5% CF + 0.15% MWCNT, (**e**) 0.75% CF + 0.1% MWCNT and (**f)** 0.75% CF + 0.15% MWCNT.

Figure 18 shows the FCR-load correlation for hybrid cementitious composites containing MWCNTs. It is clear that the correlation was much better with a lower scatter of data for these composites when compared to only CF-based and CNF-based hybrid cementitious composites. It can also be observed that the composites with 0.1% MWCNT and 0.5% CF showed a good linear correlation along with high stress sensitivity (11.82%/MPa), as observed in Figure 15 and therefore, these composites can be considered as the best CNT-based hybrid cementitious sensors for the sensing of stresses in civil engineering structures.

As discussed earlier, the presence of CNFs and MWCNTs in CF-based cementitious composites increased the conductive pathways preventing random and abrupt changes in the electrical network under tensile loading. Therefore, a more stable, accurate and reliable piezoresistive behavior was obtained in the presence of MWCNTs/CNFs. The results were superior with MWCNTs at low concentrations when compared to CNFs, due to the higher electrical conductivity of MWCNTs and owing to their superior ability to form percolating and piezoresistive electrical networks when dispersed homogeneously, due to their smaller dimensions. It can be noticed in Figure 17 and Figure 18 that the slopes of the FCR-load curves changed with the loading rates, indicating that the stress sensitivity was dependent on the loading rates. As discussed in Section 3.4.2, the loading rate could influence the tensile strain of the developed cementitious composites and this, in turn, influenced the change in the electrical resistivity. A higher loading rate was expected to reduce the tensile strain, thereby reducing the change in the electrical resistivity and stress sensitivity of the composites. However, in the present study, the applied load was also changed along with the loading rates, and this resulted in an opposite effect on stress sensitivity, i.e., an increase in the load could increase the tensile strain and increase the stress sensitivity. Due to this reason, the effect of the loading rate on the stress sensitivity of the developed composites was not very clear in the present study and needs to be further investigated.

### 3.6. Microstructure of Developed Cementitious Composites

**Figure 19:**
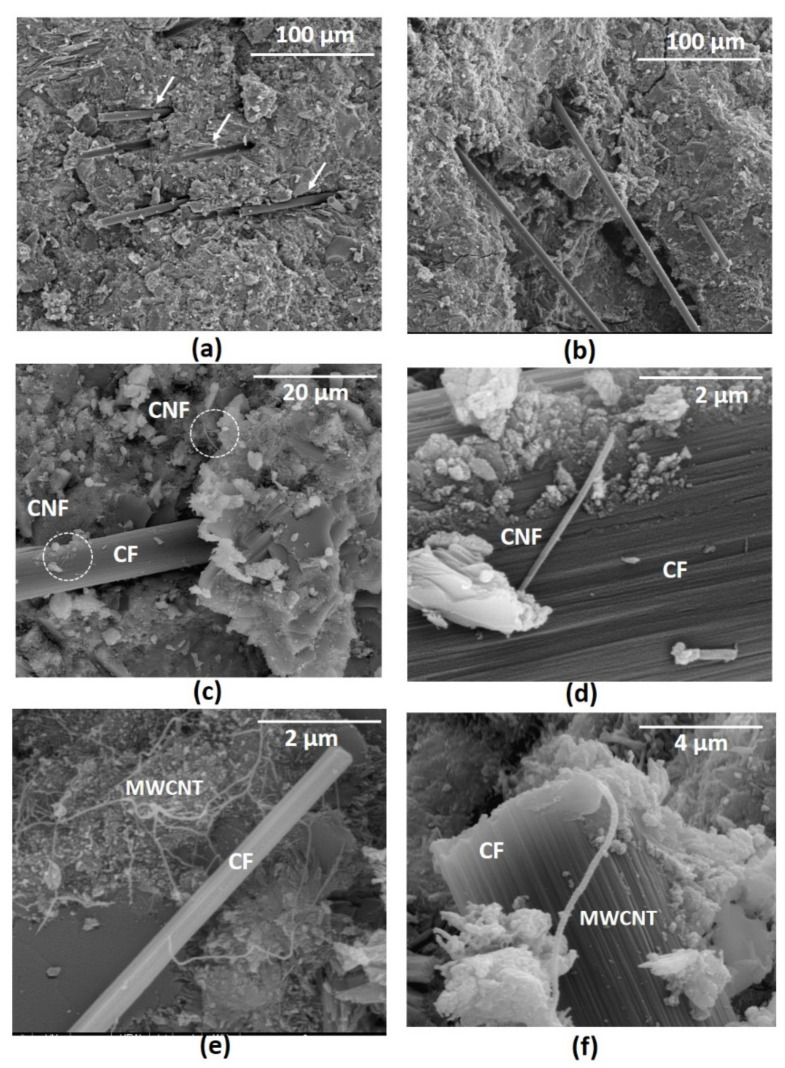
Fracture surface of a plain mortar (**a**,**b**), cementitious composite with CNFs (**c**,**d**) and a cementitious composite with MWCNTs (**e**,**f**) at different magnifications.

The fracture surfaces of broken samples in tensile tests (up to failure) were studied using SEM and are shown in Figure 19. It can be clearly observed from Figure 19a,b that CFs (indicated by arrows) were uniformly dispersed within cementitious composites. This confirmed that the used dispersion process using magnetic stirring along with Pluronic F-127 surfactant was able to ensure the homogeneous dispersion of CFs. CNFs and MWCNTs can also be observed in the fracture surface of hybrid composites, as can be seen in Figure 19c–f. It is interesting to note from Figure 19d–f that both CNFs and MWCNTs formed electrical connections between CFs and cement hydration products and helped to form the percolating and piezoresistive networks, as discussed earlier. The SEM micrographs of fracture surfaces support the mechanism of piezoresistivity, as illustrated in Figure 14.

## 4. Conclusions

In this research, multi-scale cementitious composites were developed using CFs along with CNFs or MWCNTs and their stress-sensing behavior was characterized and compared with CF-based cementitious composites. The following conclusions can be made from the present study:

(1) The electrical resistivity of CF-based composites decreased with the increase in CF content from 0.25 to 0.75%. The incorporation of CNF and MWCNT (0.1 and 0.15% of cement weight) in CF-based composites led to a significant decrease in the electrical resistivity of cementitious composites.
(2) CF-based cementitious composites showed a reversible increase in electrical resistivity with the cyclic tensile load. The highest value of FCR was achieved at the lower CF content, i.e., 0.25% and an increase in the CF content resulted in a decrease in FCR due to the saturation in the electrical contact points reducing the stress sensitivity of the composites.
(3) Multi-scale cementitious composites also showed a reversible increase in the electrical resistivity with tensile loads. Overall, the multi-scale composites showed a lower FCR when compared to CF-based composites, due to an increase in the electrically conducting pathways. MWCNTs and CNFs formed connections between well-dispersed CFs and cement hydration products, forming a well-connected percolation network.
(4) Although CF-based composites presented good stress sensitivity, the FCR-load correlation was not good and a high scatter in FCR values was noticed. This makes the CF-based cement sensors not suitable for accurately measuring tensile stresses in practical applications. On the contrary, the multi-scale composite sensors showed a good linear correlation between FCR and tensile loads with a low scatter of data. Superior results were obtained in the case of MWCNT-based multi-scale composites when compared to the CNF-based composites. The best CNF- and MWCNT-based sensors provided stress sensitivity of 6.36 and 11.82%/MPa, respectively.

## Figures and Tables

**Table 1:** Composition and properties of cement used in the present study.

	Composition	95–100% Clinker + 0–5% Minor Additional Components
**Ordinary Portland Cement** **(CEM I 42.5 R) ^1^**	Loss on ignition	≤5%
	Insoluble residue	≤5%
	Sulphur trioxide (SO_3_)	≤4.0%
	Chloride (Cl^−^)	≤0.1%
	Initial setting time	≥60 min
	Soundness	≤10 mm
	2 days compressive strength	≥20.0 MPa
	28 days compressive strength	≥42.5 MPa ≤ 62.5 MPa

^1^ Source:www.secil.pt, accessed on 12 January 2013, Lisbon, Portugal

**Table 2:** Properties of carbon fibers, CNFs, MWCNTs, Pluronic F-127 and defoamer.

Materials	Physical and Mechanical Properties				
Tenax-e HTA40 E13 6k 400 tex	Tensile Strength (MPa)	Tensile Modulus (GPa)	Elongation at Break (%)	Filament Diameter(µm)	Density(g/cm^3^)
	4100	240	1.7	7	1.77
	**Physical Properties**				
	Diameter (nm)		Length (µm)	Surface Area (m^2^/g)	Purity (%)
	Inside	Outside			
MWCNT ^1^	2–5	˂8	10–30	350–420	>95%
CNF		200–600 nm	5–50 μm	>18	>70 wt.%, Ash: <5 wt.%
Pluronic F-127	Non-ionic surfactant, molecular weight: 12,500 g/mol, CMC: 950–1000 ppm				

^1^ Source: Nanostructured & Amorphous Materials, Inc, Katy, TX, USA.

**Table 3:** Composition of different samples prepared for piezoresistive characterization (each sample contains 900 g of cement).

Samples	CF (wt.% of Cement)	CNF (wt.% of Cement)	MWCNT (wt.% of Cement)
Plain Mortar	0	0	0
0.25 CF	0.25	0	0
0.5% CF	0.50	0	0
0.75% CF	0.75	0	0
0.25% CF 0.1% CNF	0.25	0.10	0
0.5% CF 0.1% CNF	0.50	0.10	0
0.75% CF 0.1% CNF	0.75	0.10	0
0.25% CF 0.15% CNF	0.25	0.15	0
0.5% CF 0.15% CNF	0.50	0.15	0
0.75% CF 0.15% CNF	0.75	0.15	0
0.25% CF 0.1% CNT	0.25	0	0.10
0.5% CF 0.1% CNT	0.50	0	0.10
0.75% CF 0.1% CNT	0.75	0	0.10
0.25% CF 0.15% CNT	0.25	0	0.15
0.5% CF 0.15% CNT	0.50	0	0.15
0.75% CF 0.15% CNT	0.75	0	0.15

**Table 4:** Fractional resistance change of composites containing CF.

Samples	Loading Rate (N.s^−1^), Max Load (N)	FCR
Mortar with 0.25% CF	20, 200	0.177
	30, 300	0.165
	40, 400	0.148
Mortar with 0.5% CF	20, 200	0.042
	30, 300	0.082
	40, 400	0.231
Mortar with 0.75% CF	20, 200	0.074
	30, 300	0.045
	40, 400	0.097

**Table 5:** Fractional resistance change of composites containing CF along with CNF/MWCNT.

Samples	Loading Rate (N.s^−1^), Max. Load (N)	FCR	
		CNF	MWCNT
Mortar + 0.25% CF + 0.1% CNF/MWCNT	20, 200 N	0.003	0.030
	30, 300 N	0.004	0.029
	40, 400 N	0.017	0.029
Mortar+ 0.25% CF + 0.15% CNF/MWCNT	20, 200 N	0.004	0.015
	30, 300 N	0.009	0.033
	40, 400 N	0.015	0.011
Mortar + 0.5% CF + 0.1% CNF/MWCNT	20, 200 N	0.010	0.039
	30, 300 N	0.008	0.038
	40, 400 N	0.008	0.028
Mortar + 0.5% CF + 0.15% CNF/MWCNT	20, 200 N	0.005	0.003
	30, 300 N	0.010	0.003
	40, 400 N	0.017	0.002
Mortar + 0.75%CF + 0.1% CNF/MWCNT	20, 200 N	0.013	0.008
	30, 300 N	0.010	0.007
	40, 400 N	0.008	0.006
Mortar + 0.75%CF + 0.15% CNF/MWCNT	20, 200 N	0.021	0.002
	30, 300 N	0.028	0.002
	40, 400 N	0.037	0.005

## Data Availability

The raw/processed data required to reproduce these findings cannot be shared at this time as the data also forms part of an ongoing study. The relevant data can be made available on request.
